# Susac syndrome (Retino-cochleo-cerebral vasculitis), the ophthalmologist in the role of the whistleblower

**DOI:** 10.1186/s12348-020-00217-z

**Published:** 2020-10-30

**Authors:** Ioannis Papasavvas, Barbara Teuchner, Carl Peter Herbort

**Affiliations:** 1 Retinal and Inflammatory Eye Diseases, Centre for Ophthalmic Specialized Care (COS), Clinic Montchoisi Teaching Centre, Lausanne, Switzerland; 2 Department of Ophthalmology, University of Innsbruck, Innsbruck, Austria

**Keywords:** Susac syndrome, Branch retinal artery occlusion (BRAO), Fluorescein angiography, Indocyanine green angiography

## Abstract

**Background/purpose:**

Susac syndrome is a rare microangiopathy of suspected autoimmune origin affecting arteries of the retina, the cochlea and the brain. The aim of the study was to give a review of the disease entity and determine the proportion of cases and their characteristics in a uveitis referral centre.

**Patients and methods:**

Charts of patients with the diagnosis of Susac syndrome seen in the Uveitis Clinic of the Centre for Ophthalmic Specialised Care (COS), Lausanne, Switzerland were reviewed retrospectively to determine the frequency of such cases in a uveitis referral centre. Clinical symptoms and signs, functional data, imaging signs and evolution were analysed in the 3 COS cases and one case shared with the Uveitis Clinic of the Department of Ophthalmology, University of Innsbruck, Austria. Characteristic signs were searched possibly allowing a prompt diagnosis.

**Results:**

During the period from 1994 to 2019 (24 years, 2045 patients), 3 charts with the diagnosis of Susac syndrome were found (0.15%). The whole collective, including the additional case, comprised three women aged 28, 32 and 63 at presentation and one man, aged 42. None of the 3 cases that were referred were diagnosed beforehand. The characteristic item found in all 4 cases was the abrupt arterial stop or segmental interruption of arteries and increased staining of arterial wall on angiography more clearly shown on indocyanine green angiography that can potentially be proposed as a crucial diagnostic element. All 4 cases responded to dual steroidal and non-steroidal immunosuppression. Under treatment, all four patients did not show any further evolution.

**Conclusion:**

Susac syndrome is a multilocation arteritis of the head that can involve the eye, ear and brain often first diagnosed by the ophthalmologist. The diagnosis is rapidly reached in uveitis referral centres but seems to be missed otherwise, A helpful angiographic sign to be searched is an abrupt or segmental arterial stop and increased staining of the arterial wall more clearly seen on indocyanine green angiography. Patients often present first to the ophthalmologist who should be acting as a whistleblower to avoid severe involvement of the brain.

## Introduction, background and aim of study

Susac syndrome (SS) is a rare occlusive microangiopathy (vasculitis) of unknown aetiology and mechanism involving arteries of the retina, cochlea and brain [1]. The syndrome is named after John O. Susac who was the first who described the disease in 1979 [2, 3]. It is characterized by a clinical triad of visual disturbances due to branch retinal artery occlusion (BRAO), hearing loss and encephalopathy. The exact prevalence is unknown but up to date slightly more than 300 cases have been published worldwide [4]. It is presumed to be an autoimmune-mediated endotheliopathy affecting the vessels of the retina, the cochlea and the brain causing ischemic infarcts in these organs [2]. These microinfarcts are leading to the typical clinical triad [5]. Recently anti-endothelial cell antibodies (AECA) were detected in 25% of the patients supporting the hypothesis of an autoimmunity targeting the microvasculature [6, 7]. It has been shown recently that CD8+ T cell-mediated endotheliopathy is the mechanism of arterial wall inflammation in Susac syndrome that can be blocked by anti-α4 integrin monoclonal antibodies [8]. Eyes obtained at autopsy from patients with SS confirmed in histopathological examination artery occlusion at side of endothelial cell dysfunction and glia also seems to be involved [9]. The blood vessels often lacked viable endothelial cells, the wall of the arteries appeared thickened with amorphous material and dome shaped serous like material was located below the internal limiting membrane [9].

### Clinical presentation

#### Ophthalmic findings

At least 50% of patients have visual disturbances as first clinical manifestation [4]. Patients complain about reduced visual acuity, scintillating scotomas, photopsia or visual field defects. The characteristic fundoscopic findings in patients with SS are branch retinal artery occlusion or arterial narrowing and small punctuate yellow-white arterial wall plaques; these plaques are also called Gass plaques [10] and can resolve overtime [10, 11]. The findings in retinal fluorescein angiography (FA) are pathognomonic and show segmental arteriolar wall hyperfluorescence (AWH) with dye leakage in 96% of the patients [4], often occurring in a multifocal fashion and located distant to areas of branch retinal artery occlusion (BRAO). Moreover, non-perfused retinal arterioles or arterial luminal narrowing with a preserved downstream blood perfusion can be found in FA. This arterial mural staining indicating an impaired integrity of the arterial or arteriolar wall may be found unilaterally or bilaterally [10]. A progression of the AWH into BRAO has been documented in some cases but it is unclear why some AWH result in BRAO and others do not. It is important to know that AWH and arterial luminal narrowing in FA can even be found in a normal appearing fundus [12]. Indocyanine green angiography (ICGA) is showing hypofluorescence in the areas of retinal infarction and is also showing retinal vessel abnormality while choroidal circulation appears as normal [13]. Optical coherence tomography (OCT) has recently become a valuable diagnostic tool. In a case series, 68% of SS eyes showed significantly reduced average retinal nerve fibre layer thickness (RNFLT)). Characteristic is the very distinct pattern of patchy thinning of the inner retina while the outer retina remains normal reflecting arterial distribution [14]. In OCT sectors with severe inner retinal thinning are located adjacent to normal appearing sectors [12, 14]. OCT provides complementary diagnostic information to FA especially in chronic or later stages of the disease.

#### CNS manifestation

The most common clinical manifestation at onset of SS is encephalopathy (two thirds of patients) [4]. The symptoms are headache, cognitive impairment, changes in personality, sensory and motor disturbances, ataxia and confusion. On MRI involvement of the corpus callosum with typical small multifocal snowball-like lesions in T2 weighted images can be found in 78% in the acute phase and are considered as a characteristic sign of SS [15]. Moreover T2 weighted images show supratentorial white matter lesions in 98% and T2 or FLAIR hyperintense lesions in 70–100% in the periventricular white matter, subcortically, and in the deep grey matter nuclei [15, 16]. The central callosal lesions differ from those in demyelinating disease, which is the most important differential diagnoses. The cerebrospinal fluid shows a moderate elevation of proteins and a mild pleiocytosis. Oligoclonal bands (OCBs) can be found in about 15% of the patients differentiating SS from MS where oligoclonal OCBs can be found in up to 98%, being helpful only when OCBs are negative [17].

#### Hearing impairment

Hearing loss can occur overnight involving one ear with the second ear following within a few days. A loss of low or midtone range is typical but also a loss of high frequencies can be observed. Roaring tinnitus and vertigo are frequently accompanied with hearing loss or can precede it [1–4].

### Clinical course

The typical age at onset of the disease is between 20 and 40 years of age but the age range extends from 2.5 to 72 years [4, 18, 19]. It predominantly affects women with an estimated male/female ratio of about 1:3.

Three clinical courses can be distinguished: A monophasic, a polyphasic and a chronic continuous course. In the majority of cases (54%) SS is monophasic, predominantly with encephalopathy and often limited to 1–2 years with a good prognosis if treated early [18]. In the polyphasic course patients suffer from recurrent branch retinal artery occlusion and hearing loss over several years. The time between the relapses can be very long up to 18 years [19]. In the chronic continuous form, symptoms are fluctuating without real periods of remission.

### Differential diagnosis

The most likely differential diagnoses are inflammatory demyelinating CNS diseases like multiple sclerosis (MS) and acute disseminating encephalomyelitis (ADEM). Other important differential diagnoses are retinal vasculitis with or without systemic disease [20].

### Treatment

As SS is a rare disease no randomized controlled trials have been published and treatment strategies vary considerable and are based on the results of case series. Based on the hypothesis of being an autoimmune disease treatment has to be immunosuppressive. As first line high dose iv. corticosteroids are recommended with methyprednisolone (500 - 1000 mg/d) for 3 days followed by oral dose of 1 mg/kg per day for the first 2 to 4 weeks; tapering is depending on the clinical picture and can be between 10 and 20% every 2 weeks [21–24]. To reduce corticosteroids immunosuppressive agents like mycophenolate mofetil, azathioprine or cyclosporine, should be added early [22, 25]. Other possible treatment options are plasma exchange or the application of subcutaneous immunoglobulins (sc IgG) [26]. Treatment with monoclonal antibodies (Rituximab) or tumor necrosis factor (TNF) inhibitor Infliximab have been described [27, 28]. In addition to reduce the risk of microvascular thrombosis and vascular occlusion, treatment with antiplatelet agents and antivasosplastic agents can be considered [2, 24].

## Aim

The aim of this report was to analyse the ophthalmic clinical and imaging signs of SS in four patients seen in an ophthalmology referral centre. Characteristic features were sought that, when present, would raise the awareness of the ophthalmologist to the diagnosis of SS and so avoid to, mistakenly, consider these cases as common retinal vasculitis and/or ischemic events. The ophthalmologist is brought to play the role of a whistleblower in this condition in order to avoid potential severe consequences for the ear and/or the brain.

## Results

### Frequency

Among the 2045 new cases of uveitis seen at the uveitis clinic of the Centre for Ophthalmic Specialised care (COS), Lausanne, Switzerland, 3 patients were diagnosed as Susac syndrome, amounting to 0.15% of cases in a specialised uveitis centre.

### Demographics and diagnosis

The series from 2 centres analysed clinically was composed of 3 women aged 28, 32 and 63 years and one man aged 42 years. One patient directly consulted the uveitis referral centre in Innsbruck and was diagnosed without delay. One patient presented a posterior pole retinal infarction with a central scotoma and was immediately referred and diagnosed without delay. For the other two cases the diagnostic delay was 4 and 48 months once they were seen in the referral centre (Table 1).

**Table 1:**
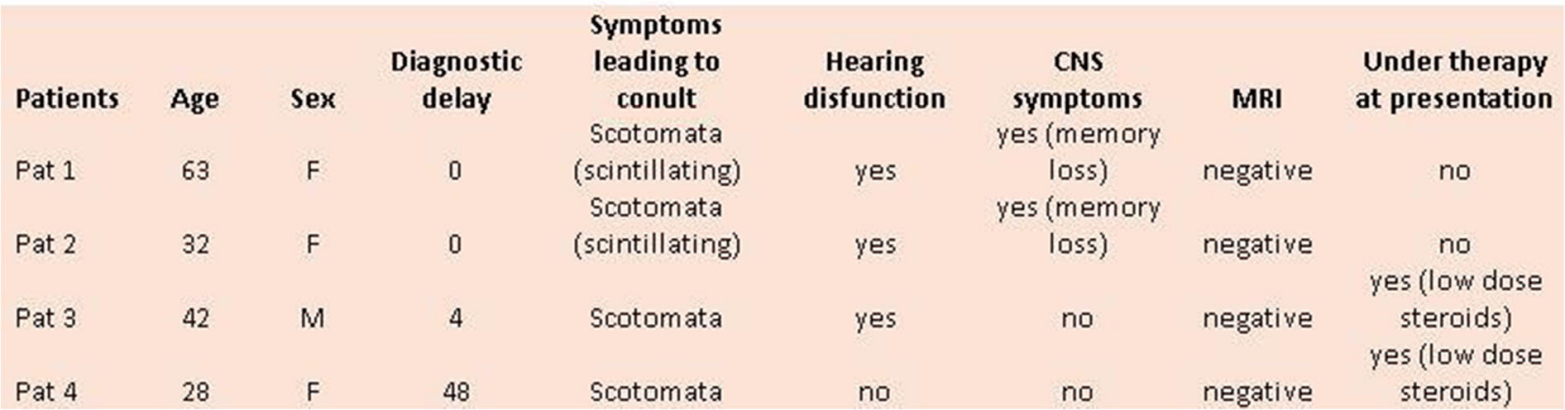
Demographic and clinical data

### Ophthalmic and systemic presenting signs

The constant ophthalmic presenting sign was a subjective scotoma that was scintillating in two cases. This was associated with hearing disturbances in 3 cases and cerebral signs such as memory loss in two cases. Two patients were under low dose systemic corticosteroid treatment, when seen in our centre.

### Ophthalmic features

Snellen chart visual acuity was 1.0 OU in the 2 patients diagnosed early and in the patient in a subacute phase. In the patient seen 48 months after the initial symptoms, VA was 0.2 OD and 0.9 OS.

There was no anterior uveitis recorded in any of the patients and laser flare photometry was normal in the two patients with initial onset disease who underwent this test.

#### Fundus

Fundus findings depended on the stage of evolution of the disease. The case seen immediately after retinal infarction had been detected by the treating ophthalmologist and showed the typical yellow-white retinal discoloration of the ischaemic zone.

**Fig. 1:**
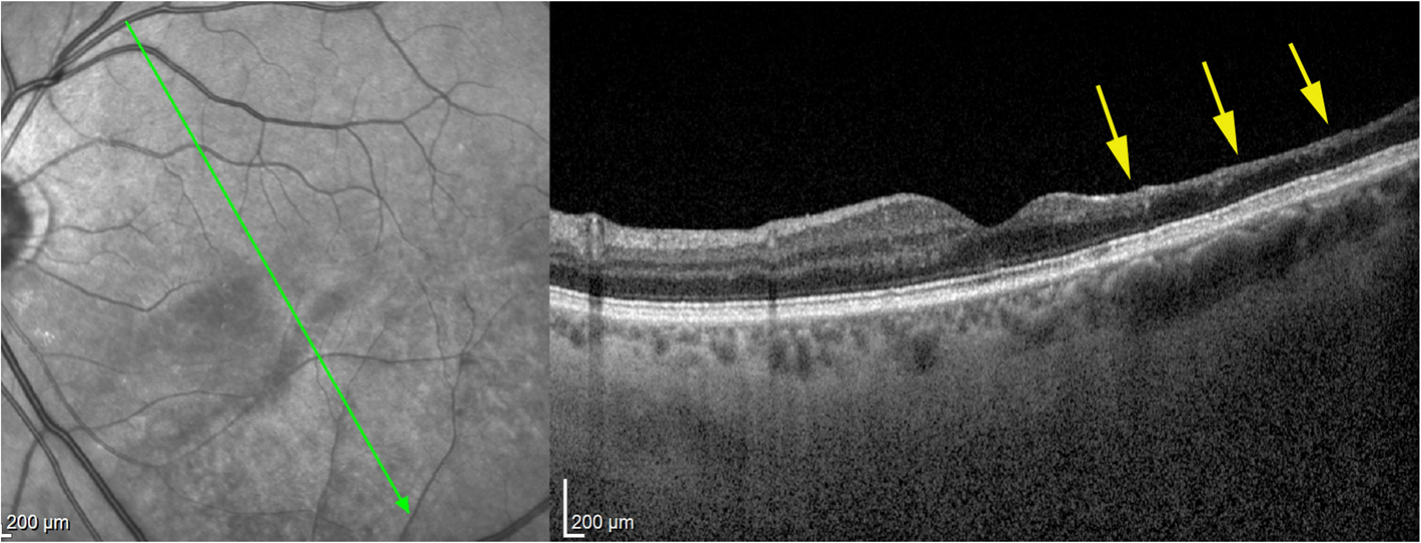
Six weeks after presentation the ischaemic zone resulted in atrophy the of the inner retina; shown by OCT imaging (arrow); note that photoreceptor outer segment ellipsoid zone is conserved (case 1)

This area had disappeared 6 weeks later and was replaced by retinal atrophy well shown on optical coherence tomography (OCT). (Fig. 1).

**Fig. 2:**
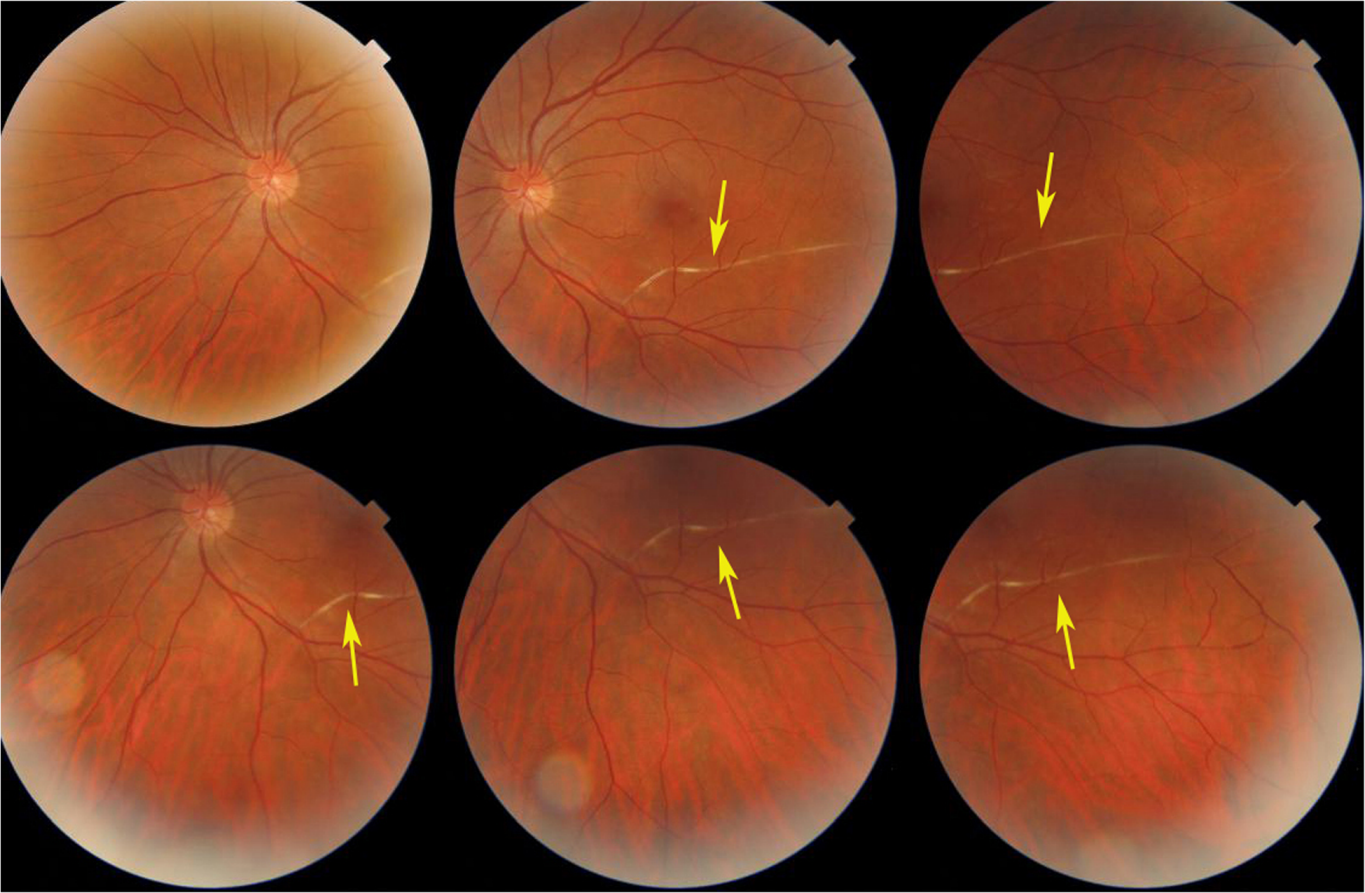
White-yellow discoloration of the course of a branch artery from inferior arcade (arrows) in a case evolving since 4 months (case 3)

For the other three patients where disease had evolved for more than 6 weeks no fundus signs of ischaemia could be detected. However, in 2 cases segments of white-yellow discoloured arteries were detected. (Fig. 2).

#### Optical coherence tomography (OCT)

**Fig. 3:**
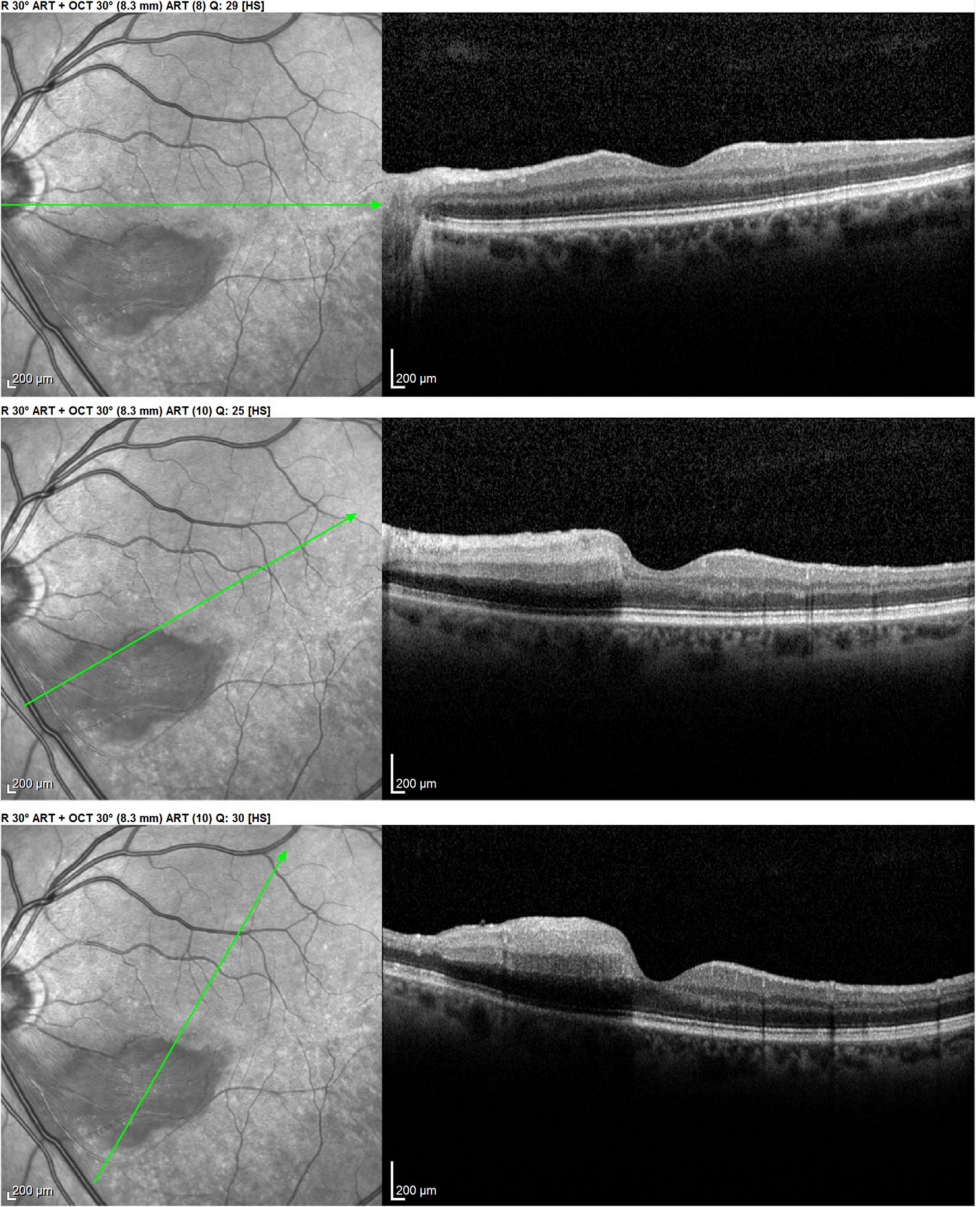
Optical Coherence Tomography (OCT) shows oedematous retinal thickening in the infarcted area in a patient seen in the acute phase (case 1) The SLO fundus image clearly delineates the ischaemic area

In the patient seen in the acute phase (patient 1), oedematous thickening of the ischaemic area could be seen at presentation (Fig. 3) evolving towards atrophic thinning of the inner retina. (Fig. 1).

**Fig. 4:**
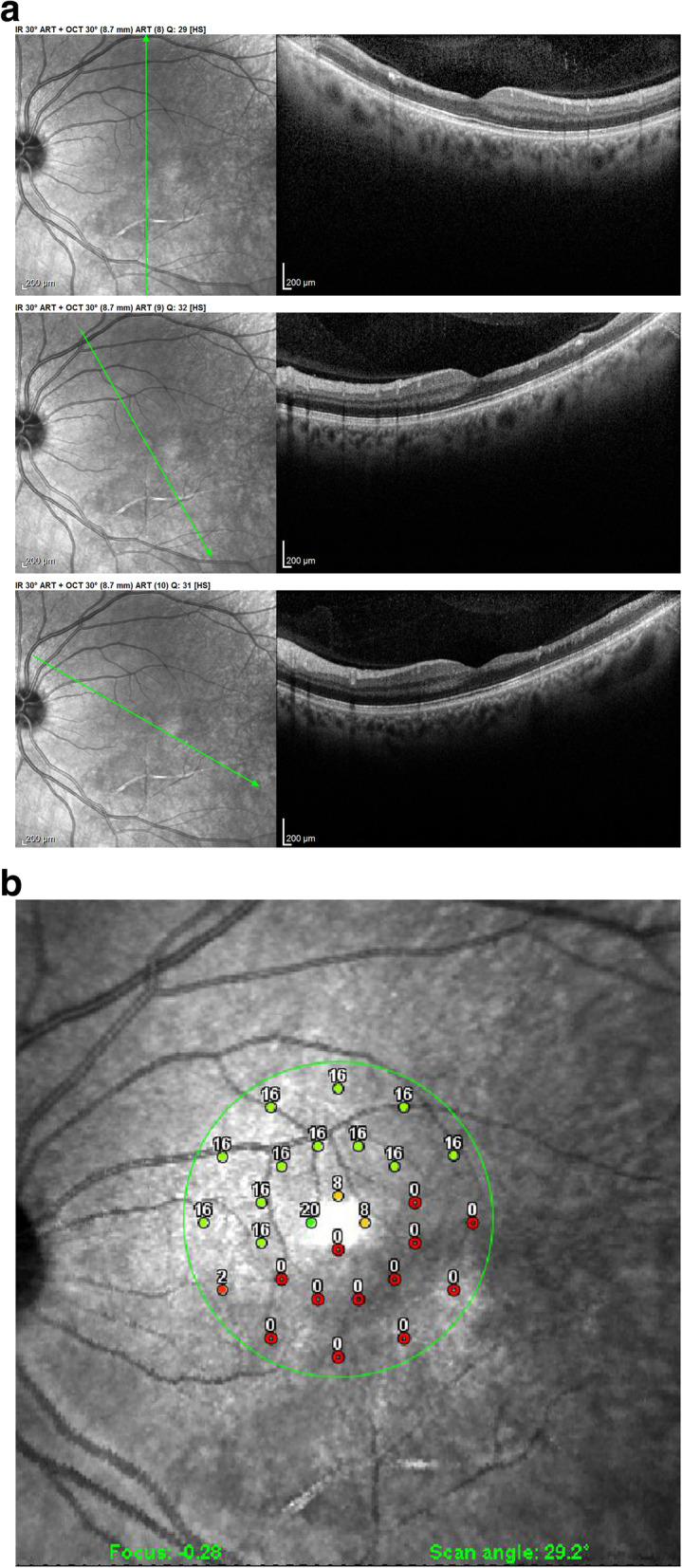
**a** OCT imaging of the retina in a patient 4 months after retinal infarct showing atrophic thinning of the inner retina with conservation of the photoreceptor outer segments ellipsoid zone; note white course of involved artery on the SLO fundus picture (case 3). **b** Microperimetry clearly delineating the non-functional atrophic retinal area (case 3)

All other 3 patients showed areas of atrophic retinal thinning. (Fig. 4 a & b)).

#### Angiography

**Fig. 5:**
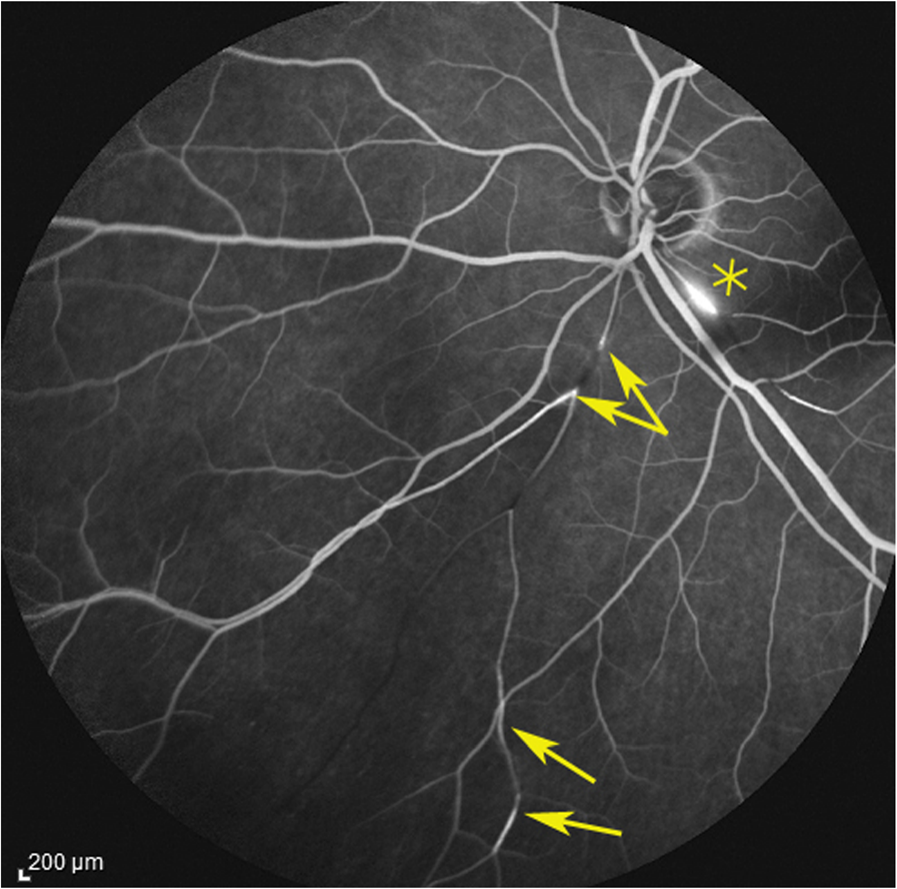
Fluorescein angiography (FA) showing abrupt interruption of two arteries inferiorly (arrows) and characteristic arterial wall hyperfluorescence (asterisk) (case 1)

**Fig. 6:**
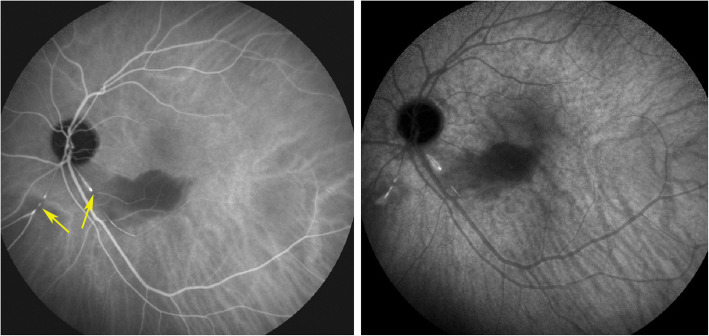
Indocyanine green angiography. Same view of fundus as on Fig. 5, showing precisely arterial stop and/or interruption (arrows) (case 1)

**Fig. 7:**
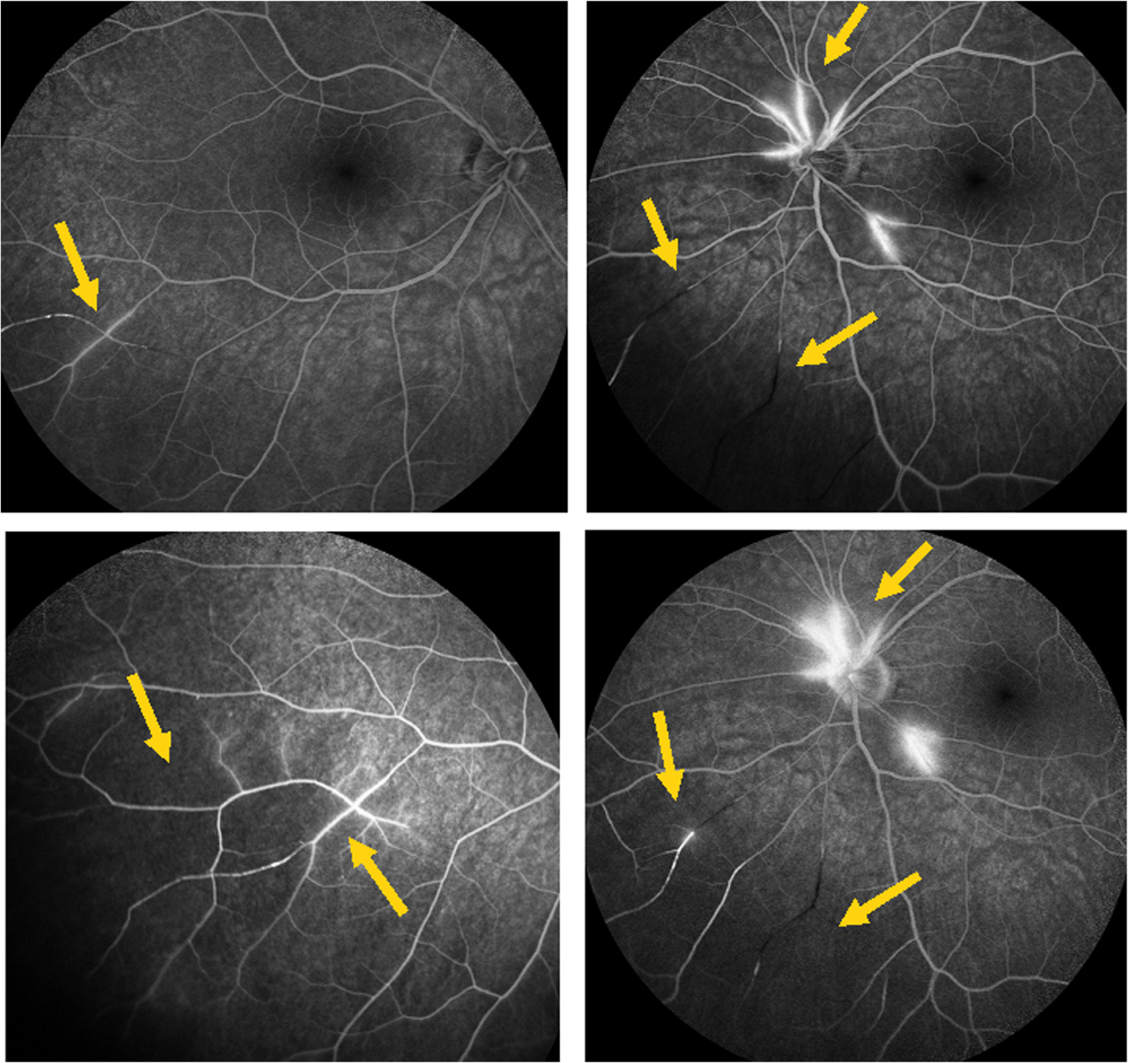
Fluorescein angiography (FA) showing segmental artery occlusion and hyperfluorescence of the arterial walls in the left eye (two right pictures) in early (top) and late (bottom) angiographic phases. In the right eye (two left pictures) an area of non-perfusion (capillary drop) of vessels in the mid-periphery (top) with staining of arteries better seen in the late phase (bottom). (Case 2)

Angiographic signs were the most characteristic and relevant features that could be determined. In all four cases an abrupt stop or segmental interruption of flow was found (Fig. 5, Fig. 6). The second sign was hyperfluorescence of the affected arteries present in all cases (Fig. 7). These two signs were more clearly visible on ICGA in the three patients for whom this investigative procedure was performed (Table 2).

**Table 2:**
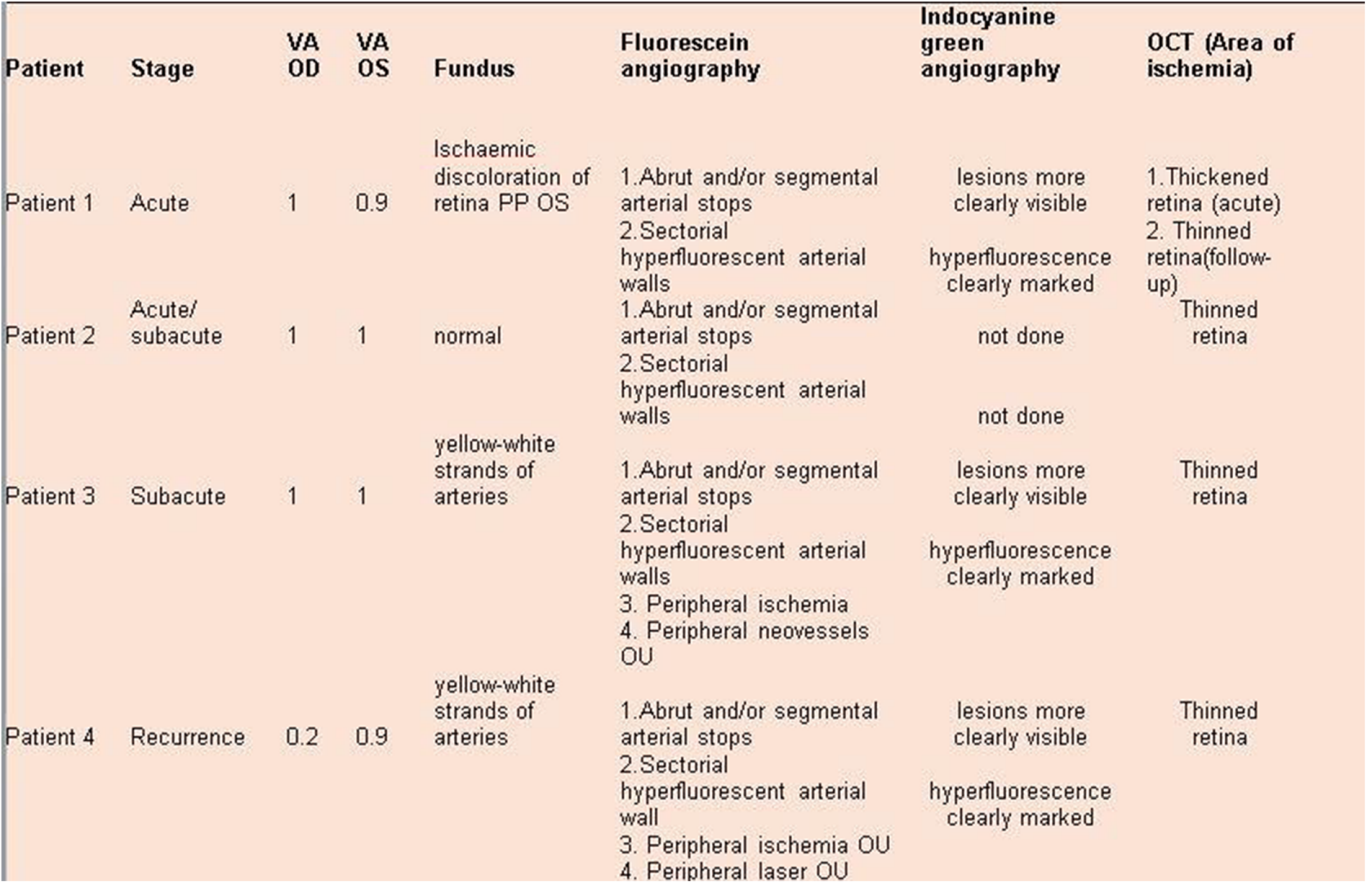
Ophthalmic, angiographic and OCT data

**Fig. 8:**
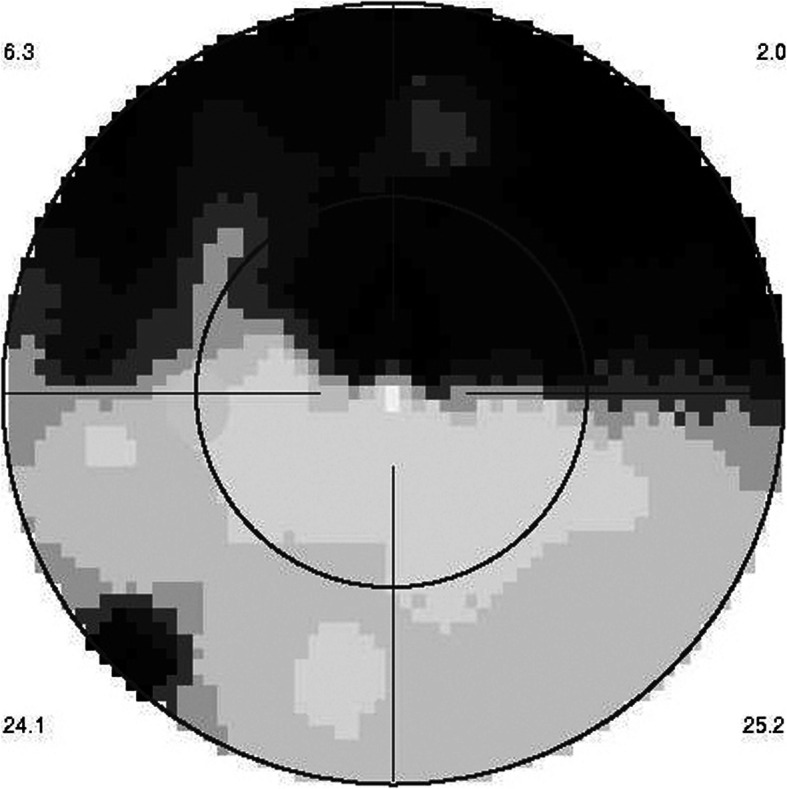
Vast upper scotoma corresponding to the ischaemic infarcted lower retina delineated by microperimetry (Fig. 9) (case 1)

**Fig. 9:**
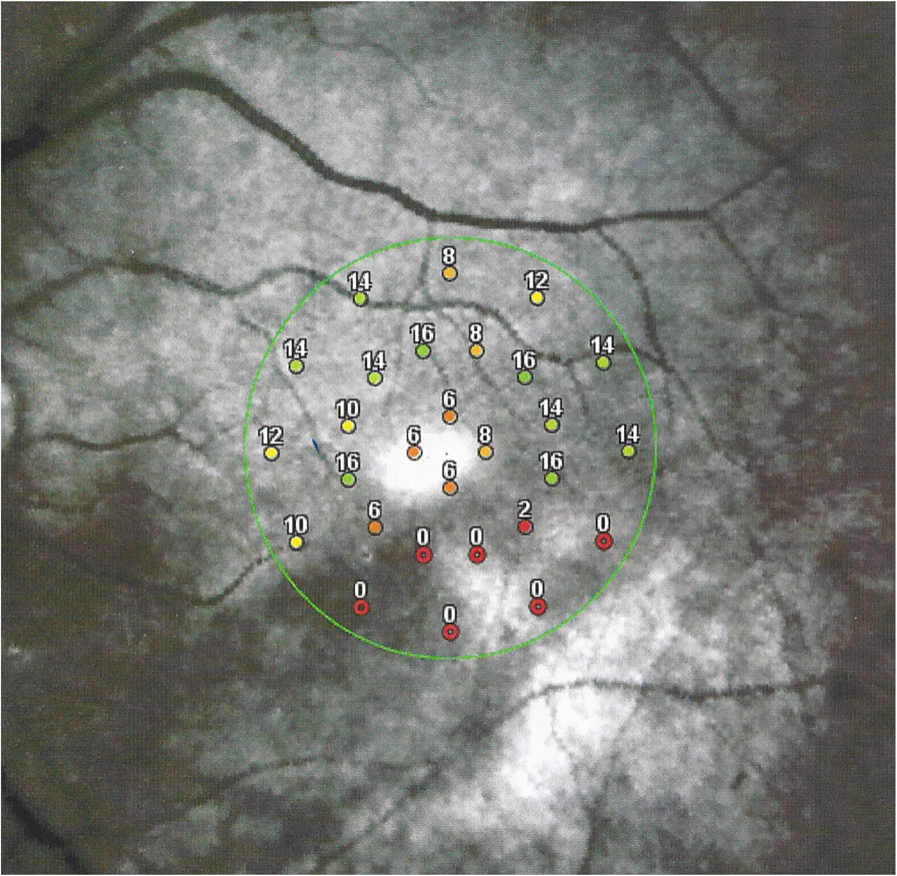
Microperimetry showing the area of functional impairment caused by the retinal infarction. (Case 1)

Scotomas were recorded in all four cases, corresponding to the areas of Retinal atrophy shown by OCT. (Fig. 8) with more precise delineation obtained by microperimetry. (Fig. 9).

### Associated investigations

MRI was negative in all four cases and a hearing loss was objectivised in 2 cases.

### Treatment

Patients 3 and 4 were under low doses of systemic steroids at presentation. Patient 1 was treated with intravenous steroids (methylprednisolone 500 mg) for 3 days with a per os relay of steroids combined with mycophenolic acid (Myfortic®,1440 mg daily). Treatment was maintained and tapered over a period of 5 years. She is now off treatment without recurrence for 9 months. Patient 2 was treated with 500 mg of intravenous methylprednisolone for 3 days followed by a combination of oral steroids and mycophenolate mofetil (Cellcept®, 2 g daily) tapered after a period of 5 years to twice 500 mg for 9 months and then discontinued The patient did not present a recurrence after a follow-up of 6 years under treatment.

Patients 3 and 4 were both treated with 500 mg of intravenous methylprednisolone for 3 days associated with cyclosporine (Sandimmun 4 mg/kg) and mycophenolate mofetil (Cellcept®, 2 g daily) tapered over 1–2 years. These two patients were lost to follow-up.

All 4 patients received anti-platelet treatment, in form of aspirin 100–300 mg daily.

### Evolution

Evolution was favourable in the two first patients who were devoid of recurrence after withdrawal of all treatment. Evolution was favourable in patients 3 and 4 but were subsequently lost for follow-up.

## Case report

**Fig. 10:**
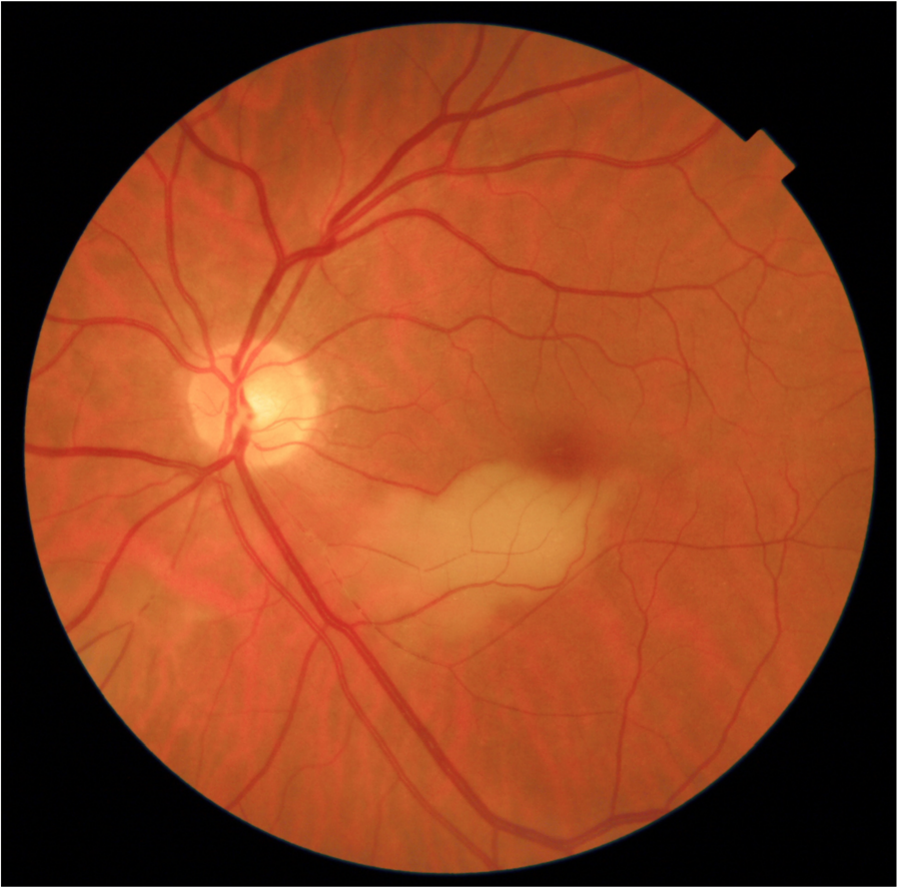
Yellow-white zone of retinal infarction (case 1)

**Fig. 11:**
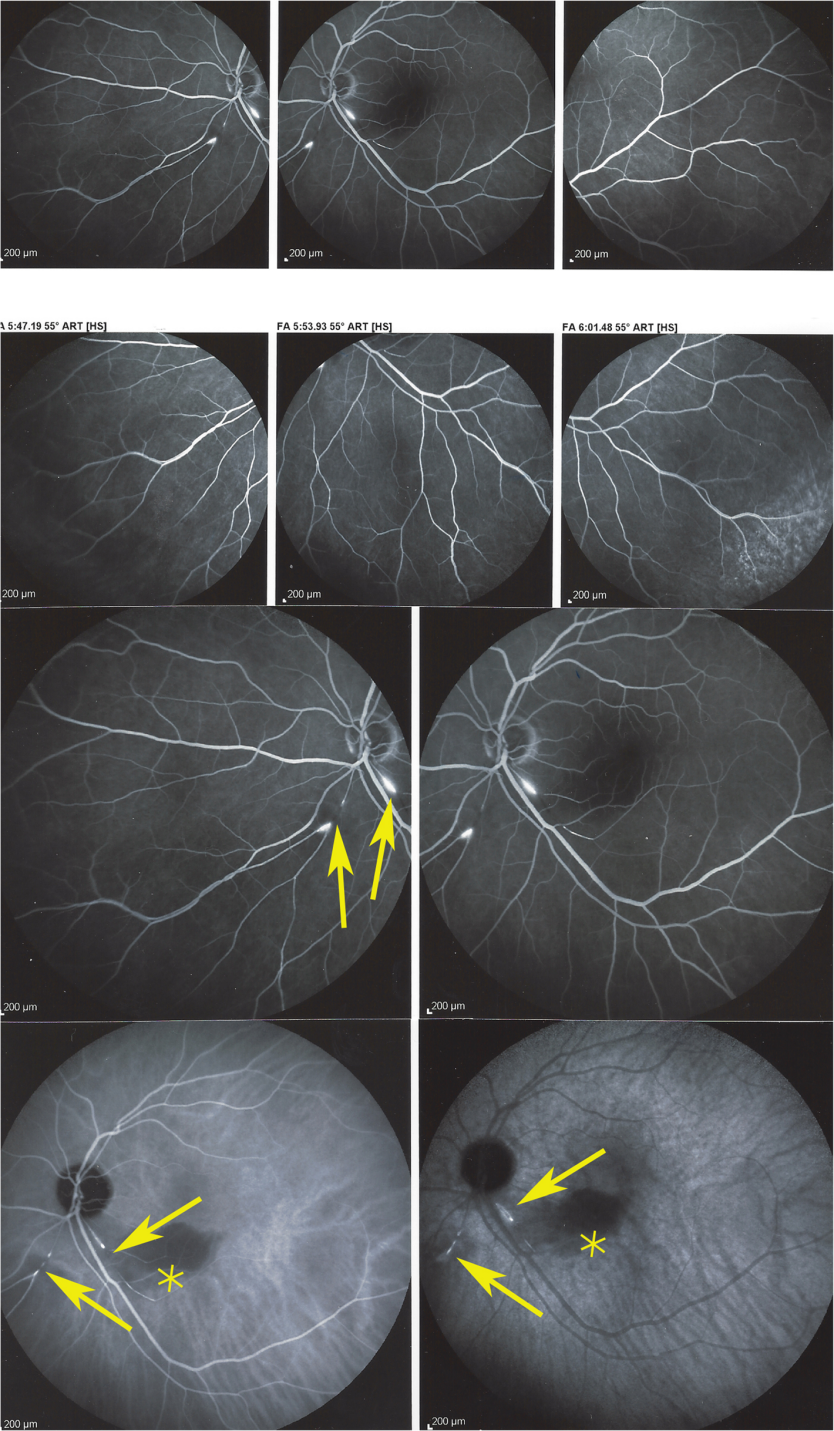
Panorama and posterior pole FA views showing arteritis with segmental involvement and abrupt artery occlusion or subocclusion (top sextet of frames). Posterior pole FA and ICGA views showing hyperfluorescence of the arteries on both FA (top two frames) and ICGA (bottom two frames) (arrows) and hypofluorescence in the areas of retinal infarction (asterisk) (bottom quartet of frames) (case 1)

**Fig. 12:**
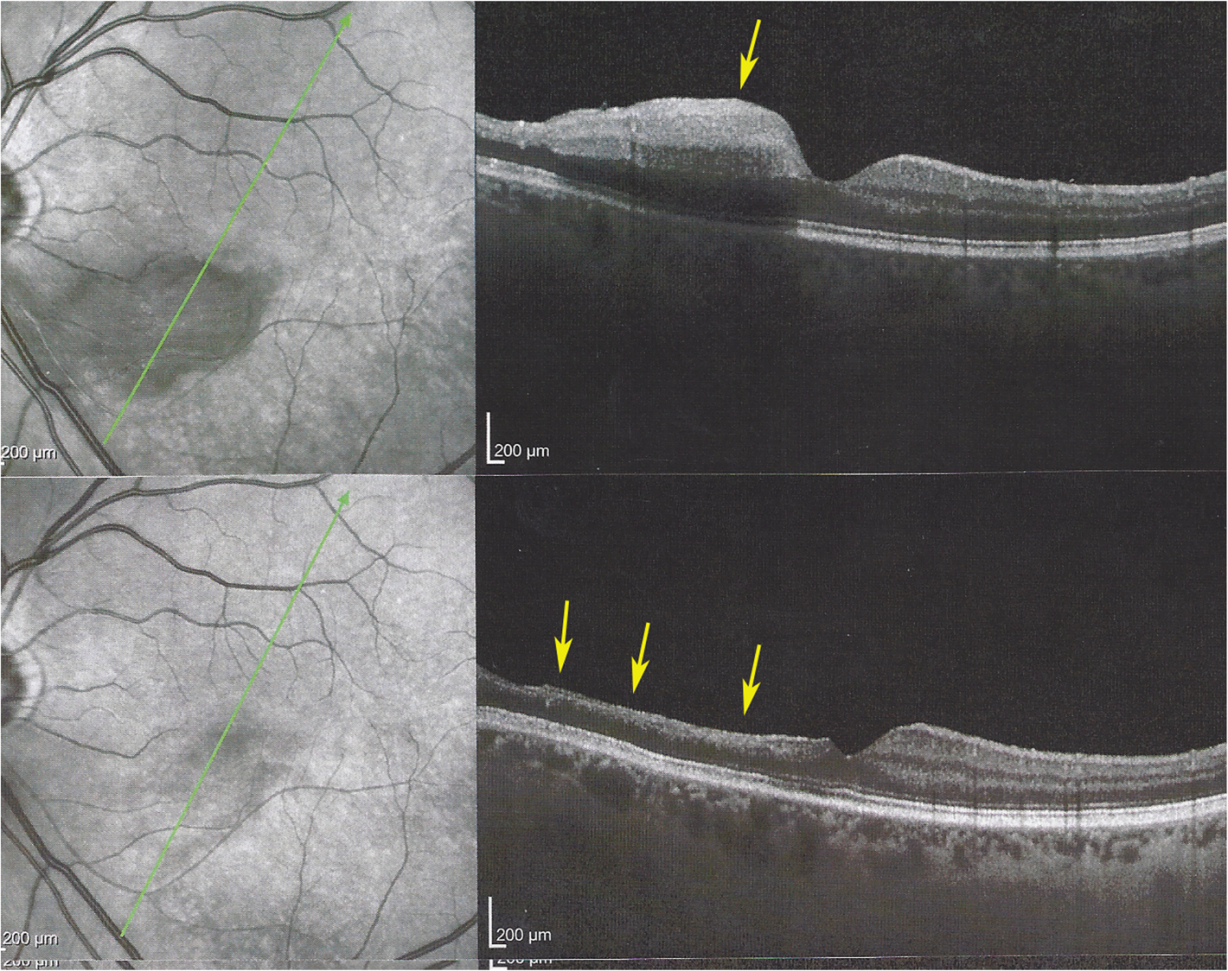
Evolution of OCT views of infarcted zone in the acute phase (top) showing retinal oedema (arrow) and 6 months later (bottom) showing atrophy of inner retina (arrow) while external retina is conserved. (case 1)

**Fig. 13:**
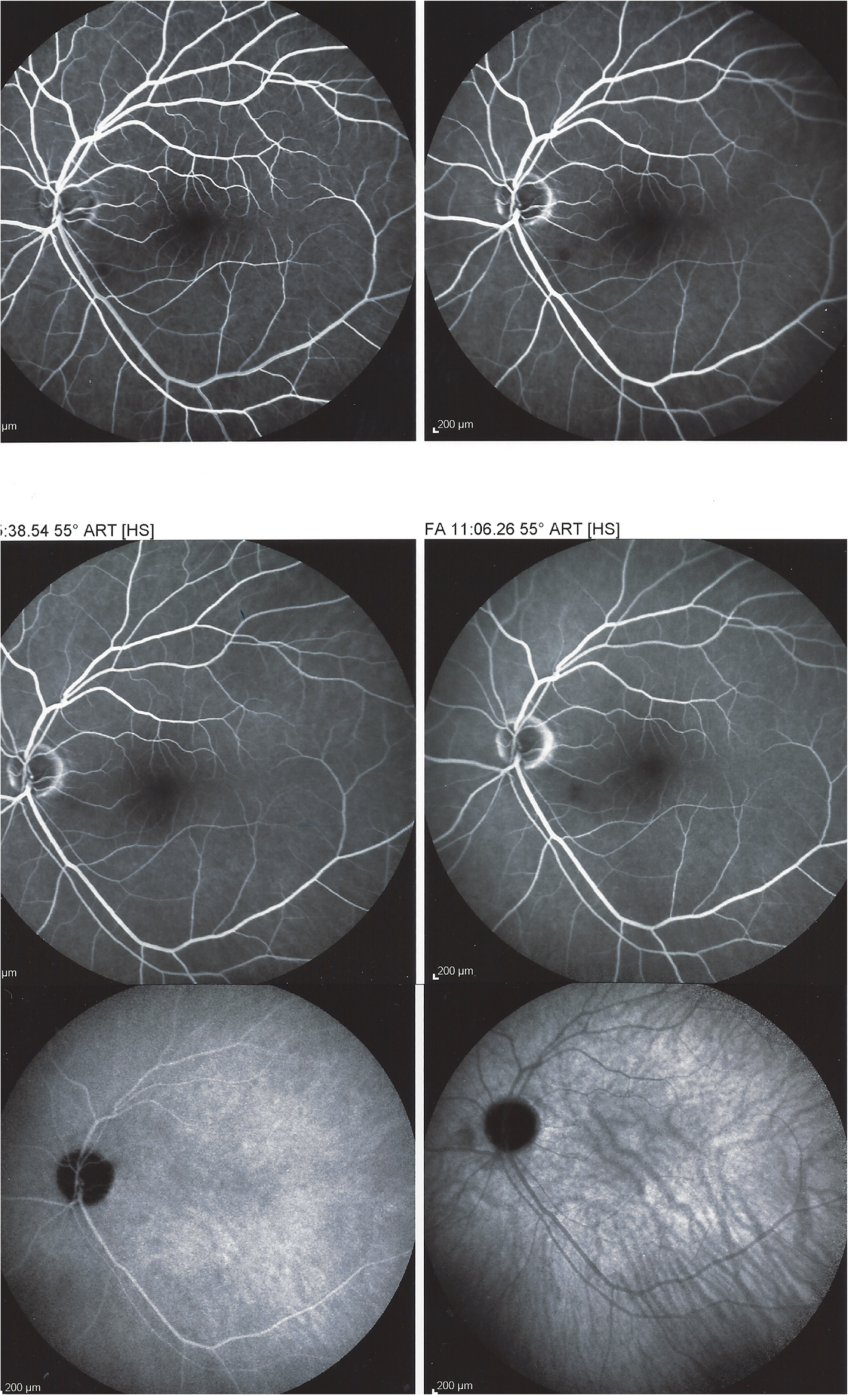
Follow-up FA (top four frames) and ICGA bottom twoframes 5 weeks after acute episode and systemic corticosteroid treatment; complete resolution of arteritis (case 1)

**Fig. 14:**
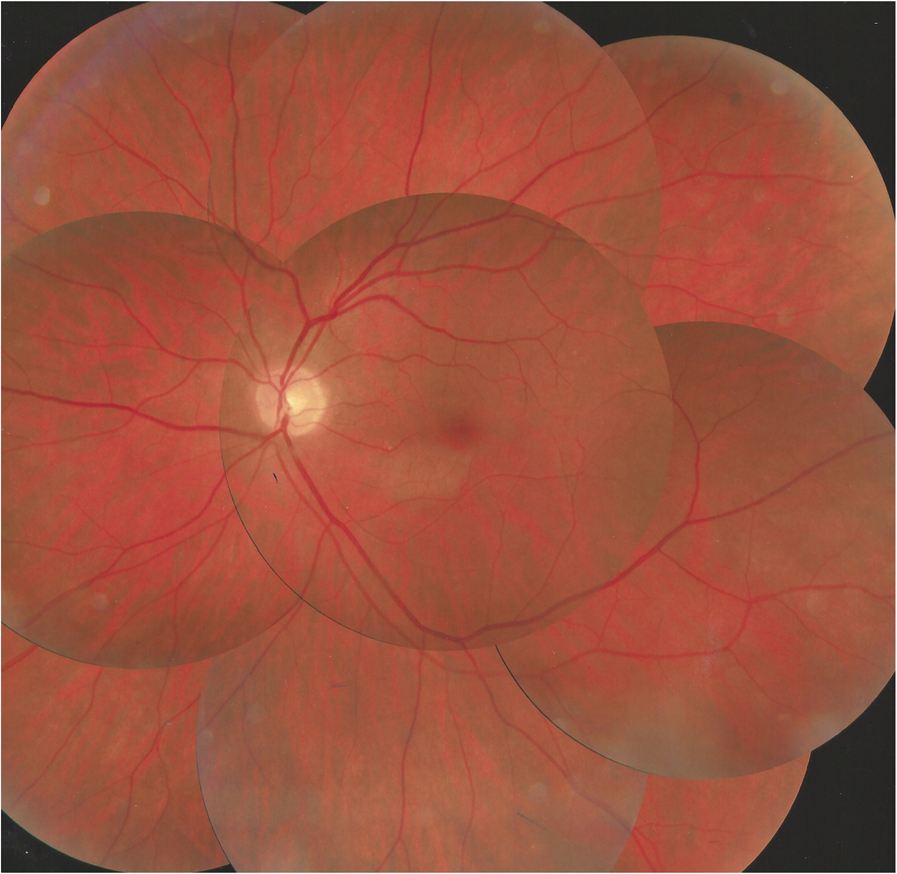
Fundus picture OS taken 5 weeks after the acute episode showing quasi disappearance of whitish aspect in the infarcted zone (case 1)

**Fig. 15:**
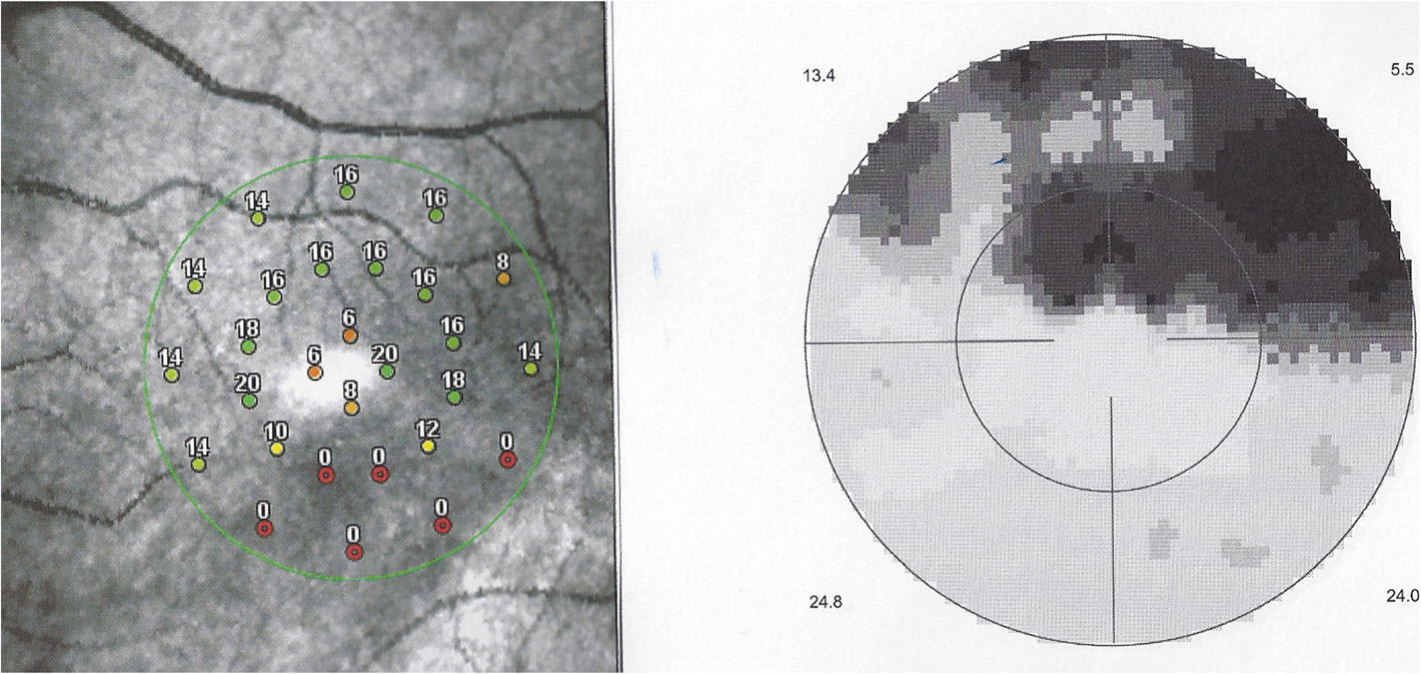
Visual field and microperimetry 6 months after the acute episode, showing improved microperimetry score (left) and slight reduction of scotoma (right) (case 1)

This 63 year old female patient woke-up one morning with a large black dot which she described as a cloud in her central visual field, accompanied by scintillations. The patient mentioned hearing difficulties on her left side and a tendency to have memory blackouts since several weeks. Except for frequent migraine episodes without ocular symptoms her history did not reveal any particular additional health problem. She consulted her eye doctor who objectivised the scotoma by Goldman perimetry and who immediately referred the patient. Best corrected visual acuity was 1.0 (OD) and 0.9 (OS). There was no anterior segment inflammation, verified by laser flare photometry, and there were no cells in the vitreous. Fundus examination showed an area of whitening of the retina situated inferiorly and nasally to the fovea. (Fig. 10) Octopus® (Haag-Streit), Bern, Switzerland) visual field testing showed superior-temporally to the fovea an absolute scotoma. (Fig. 8) and microperimetry showed an absolute and relative loss of sensitivity of the retina corresponding to the whitish area seen on the fundus photography. (Fig. 9) Fluorescein angiography (FA) showed a perfusion delay in the lower half of the retina. The striking finding however was segmental vasculitis of the arteries with in one area abrupt sub-occlusion of an artery with intense bulging exudation. (Fig. 5) On Indocyanine green angiography (ICGA) the segments of arteritis appeared also hyperfluorescent. In addition, ICGA displayed also hypofluorescence in the zone of retinal infarction that was either produced by the retinal edema and/or choriocapillaris circulation impairment due to the edema pressing on the choriocapillaris, or both. (Figs. 6 & 11). Optical coherence tomography showed a substantial retinal edema caused by retinal infarction. (Figs. 3 & 12) The neurologic examination as well as the cerebral MRI were within normal limits. Intravenous methylprednisolone was immediately introduced (500 mg per day for 3 days) followed by oral prednisone (60 mg per day) associated with mycophenolic acid (Myfortic®, 1440 mg daily).and acetylsalicylic acid (300 mg daily). After 5 weeks FA and ICGA findings reverted to normal with no arteritis and fading of the yellowish retinal infarcted area. (Figs. 13 and 14) Substantial thinning of the inner retina with conservation of the outer retina was seen on the follow-up OCT. (Fig. 12, bottom frame) Visual field and microperimetry showed a slight improvement. (Fig. 15).

Patient 1 was treated with intravenous steroids (methylprednisolone 500 mg) for 3 days with a per os relay of steroids combined with mycophenolic acid (Myfortic®,1440 mg daily). Treatment was maintained and tapered over a period of 5 years. Off treatment for 9 months of follow-up she did not show a recurrence.

## Discussion-conclusion

Susac syndrome is a vasculitis of rare occurrence making up for 0.15% of cases in a uveitis referral centre. In all our patients, it was the ocular involvement that lead the patients to consult the ophthalmologist whose responsibility it is to perform a prompt diagnosis in order to avoid cerebral and cochlear complications if they are not yet present. Our findings indicated that the diagnosis is well managed in uveitis referral centres but is missed by ophthalmologists at large.

We identified potentially disease defining findings thanks to angiographic investigation. In all patients we found constant features on fluorescein angiography consisting in abrupt and/or segmental arterial stops as well as sectorial hyperfluorescent arterial vessel walls apart from the occluded arteries. These FA signs, typical for SS were also seen on ICGA which identified these lesions more precisely. As reported earlier, we also found that ICGA showed no circulatory problems/ischemia at the level of the choroid, except a hypofluorescence in the area of the retinal infarct. We suggest to survey such findings in order to orient patients from the unspecific diagnosis of vasculitis towards the diagnosis of SS. In these cases that present ocular involvement first, the ophthalmologist’s role is crucial and acts as a whistle blower, by recognising and treating the patients and so avoiding severe cochlear and cerebral complications. Rapid identification of the disease by the ophthalmologist is crucial, as in ophthalmological series, unlike in neurological series, MRI is often negative and ophthalmic manifestations may be the sole presenting sign [29–32]. This is one more reason to have good ophthalmological disease defining criteria. In our experience all cases responded well to prompt dual steroidal and non-steroidal immunosuppression of more than 1–5 years’ duration and cerebral involvement was prevented by therapy initiated promptly in 3 patients.

Diagnosis of SS is primarily based on the clinical presentation, in particular ocular angiographic signs associated with auditory and central nervous system findings forming the classical triad. Susac syndrome is often misdiagnosed or diagnosed very late in the clinical course. One reason is the fact that the complete clinical triad is present in only about 15% at the onset of the disease [17]. The average delay between the first symptoms and the complete triad can range from some weeks to more than 2 years [4, 6]. In our patients, the time period between the disease onset and the correct clinical diagnosis was from 1 week to 126 months, as reported by other groups [6].

Susac syndrome is presumed to be an immune-mediated microangiopathy (endotheliopathy) of the retina, the cochlea and the brain. It is characterized by a clinical triad of branch retinal artery occlusion, hearing loss and encephalopathy. The treatment has to be early, aggressive and long enough, combining steroidal and non-steroidal immunosuppression to avoid visual loss, deafness and dementia.

## Data Availability

The data used during the current article are available from the corresponding author on reasonable request.
